# Milbemycin oxime and lotilaner for heartworm prevention in dogs in Korea: a randomized controlled field trial

**DOI:** 10.4142/jvs.26072

**Published:** 2026-07-14

**Authors:** Jinyeong Park, Subin Lee, Chongho Kim, Kyung-Duk Min, Hakhyun Kim, Seung-Hun Lee

**Affiliations:** College of Veterinary Medicine, Chungbuk National University, Cheongju 28644, Korea.

**Keywords:** *Dirofilaria immitis*, dirofilariasis, lactams, macrocyclic, relative biological effectiveness, safety

## Abstract

**Importance:**

Heartworm disease can lead to chronic cardiopulmonary complications in dogs, and treatment is often prolonged and costly. In Korea, *Dirofilaria immitis*, the causative agent of canine heartworm disease, has been reported in both dogs and mosquitoes and is generally considered endemic.

**Objective:**

This field study evaluated the effectiveness and safety of a combination of milbemycin oxime and lotilaner (Credelio Plus) for the prevention of heartworm disease in client-owned dogs in the Republic of Korea.

**Design/Setting/Patients:**

This randomized, single-blind, controlled field trial was conducted between June 2023 and February 2024. Client-owned dogs that were at least 8 weeks old, weighed more than 2.0 kg, were walked more than 3 times per week, and tested negative for heartworm at baseline were enrolled.

**Interventions:**

The dogs were allocated in a 1:1 ratio to receive either the investigational veterinary product (milbemycin oxime/lotilaner) or the control veterinary product (milbemycin oxime/afoxolaner) for six consecutive monthly treatments (days 0, 30, 60, 90, 120, and 150).

**Main Outcomes and Measures:**

Prevention of *D. immitis* infection was assessed monthly from days 0 to 180 using a commercial antigen test kit and conventional polymerase chain reaction. Safety was evaluated using body weight, physical examination findings, hematology and serum chemistry analyses, and adverse event monitoring to assess general health status and identify potential treatment-related abnormalities during the study period.

**Results:**

A total of 53 dogs were enrolled in the study, of which 52 were included in effectiveness and safety analyses. All dogs remained negative for *D. immitis* during the study period. In the safety evaluation, no statistically significant changes were observed in body weight, physical examination findings, or hematology and serum chemistry analyses following milbemycin oxime/lotilaner administration (*p* > 0.05). No adverse events were observed.

**Conclusions and Relevance:**

The results of this study support the use of milbemycin oxime/lotilaner combination as a safe and effective option for the prevention of canine heartworm infections under field conditions in Korea. These findings indicate that this combination product may be a useful option in veterinary parasite control.

**Trial Registration:**

Animal and Plant Quarantine Agency, Korea (clinical trial number: A221512).

## INTRODUCTION

*Dirofilaria immitis*, commonly known as heartworm, causes heartworm disease primarily in dogs, and occasionally in cats and humans [1,2]. The clinical manifestations of heartworm infection in dogs are chronic and vary with parasite burden. Prepatent infections are often asymptomatic [3]. In patent infections, adult worms primarily reside in the pulmonary arteries. However, they are also found in the right ventricles of heavily infected dogs. Associated clinical signs include fatigue, chronic cough, dyspnea, and cachexia [4,5,6,7]. In severe cases, adult worms migrate to the right side of the heart or vena cava, leading to caval syndrome, which is a life-threatening condition that can result in sudden death [8]. Managing canine heartworm disease is challenging because treatment is often prolonged, costly, and requires strict owner compliance [9]. It also has zoonotic potential and is a public health concern. In humans, *D. immitis* infection may present with various clinical manifestations, including subcutaneous nodules, chest pain, cough, wheezing, and fever [10]. Although *D. immitis* infection in humans is relatively rare, it is likely underestimated because many cases have mild or nonspecific clinical signs. Dogs serve as definitive hosts and primary reservoirs of *D. immitis*, whereas humans are accidental dead-end hosts infected by mosquito vectors [11].

*D. immitis* is a completely parasitic nematode throughout its life cycle [12]. The parasite is transmitted by several mosquito species across a wide range of genera, including *Culex*, *Aedes*, *Ochlerotatus*, *Anopheles*, *Armigeres*, and *Mansonia*, and is distributed worldwide, particularly in tropical and subtropical regions [13,14]. Owing to global warming, environmental conditions favorable to mosquito-borne transmission may contribute to an increased risk of heartworm transmission in dogs [15]. In the Republic of Korea, *D. immitis* has been reported in both dogs and mosquitoes [16], and enzyme-linked immunosorbent assay (ELISA)-based studies have reported positive rates ranging from 10.2% to 40.0% in dogs [17,18,19,20,21,22]. These regional reports support that canine heartworm infection is endemic in Korea. Human dirofilariasis caused by *D. immitis* has also been reported sporadically in Korea [23,24,25]. Given its clinical and epidemiological significance, the prevention of heartworm infection in dogs is important.

Milbemycin oxime, a macrocyclic lactone, has been widely used to treat canine intestinal nematode infections, including *Toxocara canis*, *Trichuris vulpis*, and *Ancylostoma caninum*, and to prevent heartworm infections [26,27]. In nematodes, milbemycin oxime binds to glutamate-gated chloride channels and interferes with the normal neuromuscular activity, resulting in paralysis and death [28]. Although milbemycin oxime is known to prevent heartworm infection worldwide, evaluation under field conditions in Korea is needed to support its use in dogs. Additionally, lotilaner, the other active component of the combination product, is an isoxazoline ectoparasiticide used for the control of fleas and ticks in dogs. It modulates gamma-aminobutyric acid and glutamate receptors at the arthropod neuromuscular junction, resulting in loss of neuromuscular regulation and subsequent death [29]. This field study aimed to evaluate the effectiveness and safety of a combination of milbemycin oxime and lotilaner (Credelio Plus; Elanco, USA) for the prevention of heartworm infection in client-owned dogs in Korea, compared with an approved product containing milbemycin oxime and afoxolaner (NexGard Spectra, Boehringer Ingelheim Animal Health, France).

## METHODS

This study was performed in accordance with the World Association for the Advancement of Veterinary Parasitology guidelines [30].

### Study design

This clinical investigation was a randomized, single-blind, controlled field study conducted in client-owned dogs between June 2023 and February 2024 in four regions of Korea (Gyeonggi-do, Chungcheongbuk-do, Gyeongsangbuk-do, and Gyeongsangnam-do). These regions include urban, suburban, and rural environments in which outdoor mosquito exposure may occur during the mosquito-active season.

Dogs were allocated to either the investigational veterinary product (IVP; Credelio Plus, Elanco) or the control veterinary product (CVP; NexGard Spectra, Boehringer Ingelheim Animal Health) at an approximately 1:1 ratio according to the order of enrollment. Veterinarians responsible for assessing effectiveness and safety outcomes were blinded to treatment allocation after assignment. The sample size was estimated based on non-inferiority criteria [31], resulting in a minimum of 20 dogs per treatment group. The effectiveness and safety evaluable population comprised 52 dogs, with 26 dogs in each treatment group.

Both single- and multi-dog households were eligible for enrollment, with up to three dogs per household. Dogs of any breed or sex were included and maintained under the owners’ routine management and husbandry practices. Dogs were eligible for inclusion if they were at least eight weeks of age, weighed more than 2.0 kg, were walked by their owners more than three times per week, and tested negative for *D. immitis* on day 0 using a commercial test kit (SNAP 4Dx Plus; IDEXX Laboratories Inc., USA) and conventional polymerase chain reaction (PCR). Based on owner information, clinical assessment, and available clinical records, dogs were excluded if they were pregnant or lactating, had received anthelmintics or ectoparasiticides within four weeks prior to enrollment, or had clinical conditions that could affect the study results, including malignant tumors, systemic infections, or severe hepatic or renal impairment.

### Blood sample collection

Blood samples were collected from each dog by licensed veterinarians via venipuncture into potassium ethylenediaminetetraacetic acid (EDTA) and serum separator tubes on days 0, 30, 60, 90, 120, 150, and 180. EDTA blood was used for hematology and PCR, and serum was used for serum chemistry analysis. Day 0 was defined as the first day of IVP or CVP administration. All samples were transported at 4°C to the College of Veterinary Medicine at Chungbuk National University, Korea, and stored at −20°C until further processing.

### Heartworm testing and assessment

Blood samples were screened for *D. immitis* antigens on day 0 using a commercial test kit (SNAP 4Dx Plus; IDEXX Laboratories, Inc.). Follow-up heartworm antigen testing was conducted at subsequent study visits (days 30, 60, 90, 120, 150, and 180) using the same test kit to monitor heartworm infections throughout the study.

To further confirm the presence of *D. immitis*, genomic DNA was extracted from blood samples collected on each scheduled sampling day using DNeasy Blood & Tissue Kit (Qiagen, Germany) in accordance with the manufacturer’s instructions. DNA concentration was determined using a spectrophotometer (DeNovix, USA). The extracted DNA was stored at −20°C until further use. Conventional PCR targeting the *cox1* gene of *Dirofilaria* spp. was performed as previously described [32], using the AccuPower HotStart PCR Premix Kit (Bioneer, Korea).

### Treatment

Dogs meeting all eligibility criteria, including those negative for *D. immitis* on day 0, received either IVP or CVP monthly for a total of six doses (days 0, 30, 60, 90, 120, and 150). Each subsequent dose was administered within 30 ± 3 days following the previous treatment. The IVP and CVP were available in five strengths, providing dosage ranges of 20–41 mg lotilaner/kg body weight (bw) and 0.75–1.53 mg milbemycin oxime/kg bw for the IVP, and 2.5–5.36 mg afoxolaner/kg bw and 0.50–1.07 mg milbemycin oxime/kg bw for the CVP. All treatments were administered orally with food.

### Safety evaluation

For the safety evaluation of the IVP and CVP, baseline parameters were recorded on day 0. Follow-up assessments were performed on days 30, 60, 90, 120, 150, and 180 based on body weight measurements and changes over time, physical examinations, hematology and serum chemistry analyses, and adverse event monitoring.

Dog owners were instructed to report unusual observations after IVP or CVP administration. All adverse events were recorded and evaluated for potential associations with treatment.

### Statistical methodology

A total of 52 dogs were included in the analyses of effectiveness and safety. Prevention of heartworm infection was evaluated based on a commercial test kit and PCR results obtained on days 30, 60, 90, 120, 150, and 180, after confirmation of negative *D. immitis* on day 0.

For safety evaluation, body weight changes between the IVP and CVP groups were analyzed using a linear mixed model with treatment, time, and their interaction as fixed effects [33]. Additionally, hematology and serum chemistry analyses were performed by comparing the proportion of subjects in the IVP and CVP groups whose values fell outside the reference range using a generalized linear mixed model [33]. As different analyzers were used for hematology and serum chemistry tests across clinical sites, not all parameters were available for all samples. Analyses were performed using the available data for each parameter. Statistical analyses were performed using R environment (version 4.4.2; R Foundation for Statistical Computing, Austria). Statistical significance was set at *p* < 0.05.

## RESULTS

### Dog demographics

A total of 53 dogs were included in the study, with 27 and 26 dogs allocated to the IVP and CVP groups, respectively. One dog in the IVP group died and was excluded from the study. Finally, 52 dogs (26 per group) were included in the effectiveness and safety evaluable population.

Most dogs in both groups were pure breeds, accounting for approximately 92.31% (24/26) and 96.15% (25/26) in the IVP and CVP groups, respectively. Sex distribution was similar between groups; females accounted for approximately 38.46% (10/26) of the IVP group and 42.31% (11/26) of the CVP group, whereas males accounted for 61.54% (16/26) and 57.69% (15/26), respectively. On day 0, the mean age was 78.65 ± 44.43 months in the IVP group and 76.23 ± 37.24 months in the CVP group, while the mean body weight was 7.04 ± 8.05 kg and 9.88 ± 8.94 kg, respectively (Table 1).

**Table 1:** Demographics and body weight at day 0 of dogs in the effectiveness and safety evaluable population

Variables		IVP (n = 26)	CVP (n = 26)
Breed			
	Pure	24 (92.31)	25 (96.15)
	Mixed	2 (7.69)	1 (3.85)
Sex			
	Female, intact	3 (11.54)	3 (11.54)
	Female, spayed	7 (26.92)	8 (30.77)
	Male, intact	2 (7.69)	0 (0.00)
	Male, castrated	14 (53.85)	15 (57.69)
Age (mon)			
	Mean ± SD	78.65 ± 44.43	76.23 ± 37.24
	Range	12–165	24–141
Body weight (kg)			
	Mean ± SD	7.04 ± 8.05	9.88 ± 8.94
	Range	2.00–45.00	3.20–35.80

Data are presented as number (%) unless otherwise specified.

IVP, investigational veterinary product; CVP, control veterinary product; SD, standard deviation.

### Evaluation of heartworm prevention

All 52 dogs included in the effectiveness evaluation tested negative for *D. immitis* using a commercial test kit and PCR. All the enrolled dogs received the expected dose within the dose range (0.75–1.53 mg/kg for IVP, 0.50–1.07 mg/kg for CVP).

### Safety evaluation

A total of 52 dogs were included in the safety evaluation. No statistically significant differences were observed for treatment, time, or the interaction between treatment and time in body weight analysis (*p* = 0.23, *p* = 0.80, and *p* = 0.53, respectively; Supplementary Table 1).

During the study, abnormal findings were observed in one dog (3.85%, 1/26) in the IVP group and two dogs (7.69%, 2/26) in the CVP group. In the IVP group, one dog had myxomatous mitral valve disease that persisted from day 0 until the end of the study. In the CVP group, one dog showed increased alanine aminotransferase (ALT) levels on day 180, whereas another dog exhibited gastrointestinal signs on day 116.

Statistical analysis of hematology and serum chemistry results revealed statistically significant differences between the IVP and CVP groups in the proportions of measurements outside the reference range for creatinine and platelet count (*p* = 0.04 and *p* = 0.03, respectively). These proportions were higher in the CVP group for creatinine and in the IVP group for platelet count. Four dogs in the IVP group showed persistently elevated platelet counts from days 0 to 180. No statistically significant differences were observed for the other parameters.

## DISCUSSION

Heartworm transmission can be influenced by regional differences in climate, mosquito activity, and dog management practices. Therefore, evaluation under field conditions is needed to confirm the effectiveness and safety of preventive products in the local canine population. This field study demonstrated the effectiveness and safety of a combination of milbemycin oxime and lotilaner for the prevention of heartworm infection in client-owned dogs in Korea. This study included a diverse population of dogs with a wide range of body weights, ages, and breeds, with similar sex distribution between the two groups, reflecting the general canine population under field conditions in Korea.

All dogs in both the IVP and CVP groups showed negative results for *D. immitis* using a commercial test kit and PCR during the study and received milbemycin oxime within the recommended dose range. These findings indicate that the prevention of heartworm infection by milbemycin oxime was maintained throughout the study. These results are consistent with those of previous studies demonstrating the high preventive efficacy of milbemycin oxime against *D. immitis* infections in other countries [34,35,36].

The safety profile of IVP was also evaluated. No statistically significant differences in body weight changes were observed between the IVP and CVP groups. Statistically significant differences were observed between the two groups in the proportions of measurements outside the reference range for creatinine and platelet count. However, these findings were not considered related to IVP or CVP administration because dogs with creatinine values outside the reference range showed no concurrent clinical signs suggestive of renal dysfunction, whereas the difference in platelet count was attributable to four dogs in the IVP group with persistently elevated platelet counts from days 0 to 180.

During the study, one dog in the IVP group showed pericardial effusion and cardiac tamponade on day 138 and died on day 142 despite appropriate clinical treatment. Considering the dog’s age of 9 years and 6 months, pre-existing cardiac condition, clinical progression consistent with severe cardiac disease, and the absence of gastrointestinal signs typically associated with IVP administration, such as vomiting or diarrhea [37,38], the event was assessed as unlikely to be related to IVP administration by the study researcher. Therefore, this case was excluded from the effectiveness and safety evaluable population. Among the dogs included in the safety evaluable population, one dog in the IVP group had myxomatous mitral valve disease that was present from day 0; however, this condition did not worsen during the study period, and no other adverse events related to IVP administration were observed. Therefore, this condition was considered unlikely to be treatment-related. Meanwhile, in the CVP group, one dog showed a marked increase in ALT levels on day 180, approximately nine-fold above the upper reference limit, after completing all six scheduled doses. The dog received hepatoprotective treatment; however, no additional follow-up was available because the ALT increase was detected at the final visit. Because the finding was detected only after completion of all scheduled doses and was not accompanied by clinical signs, it was assessed by the study researcher as unlikely to be related to CVP administration. Another dog in the CVP group showed intermittent vomiting and diarrhea on day 116. The dog received symptomatic treatment, and the signs resolved without further complications.

Milbemycin oxime acts on glutamate-gated chloride channels in nematodes, causing neuromuscular hyperpolarization and preventing the L3 and L4 stages of *D. immitis* from developing into adult worms [3,39]. Although reduced effectiveness of approved macrocyclic lactone-containing heartworm preventives has been reported in the United States and Italy [12,40,41,42,43,44], this has been attributed to multiple factors, including poor owner compliance, incorrect dosing, diagnostic limitations, and reduced susceptibility in some *D. immitis* strains [3,45,46,47,48]. Because related information remains limited in Korea, continued surveillance and further field studies are needed. In addition to its preventive role, milbemycin oxime at the recommended doses has been well documented in dogs, including puppies and breeding animals, with only mild and transient adverse effects occasionally reported [34,49,50]. Additionally, the IVP used in this study contained lotilaner, an isoxazoline ectoparasiticide approved for use in dogs in the US that has demonstrated rapid and sustained efficacy against fleas and ticks [51]. Although the present study was not designed to specifically assess ectoparasite control, the findings provide additional support for the safety of lotilaner administered within the recommended dose range.

The limitation of this study is that it was not possible to confirm whether each enrolled dog had been exposed to infective mosquitoes under field conditions. However, previous ELISA-based studies have reported *D. immitis* positive rates in Korean dogs from various regions [17,18,19,20,21,22], suggesting that canine heartworm is endemic in the country. In addition, only dogs that were walked more than three times per week under routine owner management were enrolled in this study, and the study period included the season when mosquito activity is common in Korea. Taken together, these factors suggest that the study was performed under conditions in which natural exposure was possible.

In conclusion, the IVP showed heartworm prevention and overall safety comparable to those of the CVP in client-owned dogs under Korean field conditions. These findings support the use of the milbemycin oxime/lotilaner combination product as a monthly heartworm preventive option in routine veterinary practice in Korea.

## SUPPLEMENTARY MATERIAL

Supplementary Table 1 Linear mixed model analysis of the effects of treatment, time, and their interaction on body weight changes
